# A Statement of the Progress in the Vaccine Inoculation, and Experiments to Obtain Determinations Concerning Some Important Facts Belonging to the Vaccine Disease

**Published:** 1799-10

**Authors:** George Pearson

**Affiliations:** Physician to St. George's Hospital


FOR THE MEDICAL AND PHYSICAL JOURNAL.

### *A Statement of the Progreſs in the Vaccine Inoculation, and Experiments to obtain Determinations concerning ſome important Facts belonging to the Vaccine Diſeaſe.* By GEORGE PEARSON, M. D. F. R. S. Phyſician to St. George's Hoſpital.

THE collection of teſtimonies which I publiſhed in November laſt in my "*Inquiry concerning the Hiſtory of the Cow-pox;*" and the circular letter which I iſſued in March laſt, ſtating the progreſs of the *Vaccine Inoculation,* and and containing thread impregnated with *matter*, have procured me much information. In particular, through the recommendation of the *Surgeon-General*, THOMAS KEATE, Efq. the new practice has been introduced into the army, of which, a valuable report has been already communicated. I have been alfo fo fortunate as to obtain permiffion to practife the *new inoculation* in certain fituations where great numbers would have been inoculated for the fmall-pox. The cafes from thefe fources, and a pretty large ftock from private practice, form a valuable body of evidence, by means of which, the profeffional public would be enabled to eftimate (I do not fay precifely) the value of the *new practice*, and alfo anfwer many of the queries, and fupply fome of the deficient parts of the hiftory of the vaccine difeafe, which are ftated in the *Inquiry* above mentioned. But fuch are my occupations at prefent, and in all likelihood, fuch they will be for a confiderable time, that I fhall be unable to arrange for the ufe of the public, the valuable materials tranfmitted to me. It will however, perhaps, be not without utility at this time, firft, to ftate a few general refults from the vaccine inoculation; and fecondly, to relate fome *trials*, from which I apprehend conclufions can warrantably be drawn, which may promote the inveftigation now going forward.

Not much more than fix months have elapfed fince the opportunity was afforded, by the breaking out of the *vaccine difeafe* in two principal milch-farms near London, of obtaining matter for propagating the fame difeafe among human creatures. The *new inoculation* was immediately introduced in London, and foon afterwards in the neighbourhood, as well as in many provincial fituations. It is with fincere fatisfaction that we can now reckon, at the feweft, 2000 perfons who have paffed through the cow-pox by inoculation; but, in this number, I include the very large proportion furnifhed by him, who, fo beneficially for the public, and honourably to himfelf, poffeffes the office of phyfician to the Small-pox Hofpital. From the above experience we receive, as I expected, important information.

1. Of the above number, it appears that *one* patient died. (Woodville's Reports, p. 151.) And to avoid controverfy, let us allow that the death was occafioned folely by the inoculation. Now, according to the jufteft calculation I have been able to make; as in the inoculated fmall-pox one in two hundred * dies from the difeafe, it is evident, in the prefent ftate of the

* I am fully aware that so great a proportion as one in two hundred, will not be allowed by many practitioners; and to persons who have been told, and believe, that inoculation for the small-pox "scarcely ever does any harm;" that certain practitioners have inoculated many thousands without losing a patient! that others have told the practice, that the proportion of fatal cases in the inoculated small-pox to the inoculated cow-pox is as ten to one.

2. The constitutional affection, or fever, which occurs in the cow-pox about the ninth day after inoculation, is much more considerable in many cases, than was apprehended from the first account by Dr. JENNER; although in a great proportion of cases it is extremely slight, and in many, cannot be observed at all. But I must correct my statement in March last, in which I said, " although the extreme cases of the severe kind which ordinarily occur in the same number of cases in the inoculated small-pox, did not occur in the new practice; and although many of the patients were even more slightly disordered constitutionally, yet the whole amount of the constitutional illness seemed to be as great as in the same number of patients in the inoculated small-pox." Since that report, or at least, for the last four months, as far as I have observed, and been able to learn from others, the whole amount of the constitutional illness was not one half of the whole amount in an equal number of patients inoculated for the small-pox. Now, whether the greater mildness of the disease depended on the different state of the human constitution in the summer from that of winter, as seems to me most probable; or that it depended on the difference in the state of the vaccine matter, must be determined by future experience in the same seasons.

3. The most remarkable difference in the practice of the last winter and present summer, has been with regard to the eruptions which so often occurred, especially in the Small-pox Hospital; which eruptions, in many instances, could not be distinguished from those of the small-pox, and which were wholly unexpected from the original description by Dr. Jenner. No explanation hitherto given, consists with the observations relative to these eruptive cases; but the facts are, as Dr. Woodville informs us they have occurred much less frequently this summer, than in the spring and winter preceding. In my private practice, not a single case with told their friends " they never had a fatal inoculated case in their whole lives:" I say to such persons, no advantage on the score of saving life will be allowed from the cow-pox; but I have conversed with many candid and experienced practitioners, and they are well satisfied that I am warranted in the above statement of deaths in the inoculated variola. I beg leave to say further, that I believe more persons in proportion have died of the inoculated small-pox within a few years, than died in the same time twenty years ago; and this may be accounted for, from the unwarrantable assertions of many inoculators; from whence a great part of the public have imbibed the opinion, that the inoculated small-pox was not attended with any danger; and the practice is often trusted in the hands of persons not sufficiently acquainted with the treatment fit for different states of the human constitution.

with eruptions resembling the small-pox, has occurred these last four months, and but a small proportion with any eruptions of other kinds. From my correspondents I have not had a single case of eruptions like the variolous, since that of Dr. REDFEARN's, of Lynn; not one of this sort in Mr. KELSON's, of Seven Oaks, report of about one hundred patients; not one in Dr. MITCHILL's, of Chatham, of about fifty patients; not one in the report of near one hundred patients from Dr. HARRISON, of Horncastle, communicated to the Right Honourable Sir JOSEPH BANKS; and, in short, not one case with these eruptions appears in the accounts from my other correspondents.

4. The arms have manifested in many instances a much more extensively spreading red areola around the inoculated part, than is usual in the small-pox, which redness sometimes extended over the greater part of the whole arm. This appearance is very alarming to both the patient and the inexperienced practitioner; but no danger seems to be attendant on such a state of the parts; for it disappears in at most two or three days; by no means gives pain in proportion to its appearance, and, in the cases I have seen, affects the constitution very little. I would rather call this spreading redness of the skin *erythema* than *erysipelas*. As to phagedenic ulcers, as they have been called, ensuing from the inoculated part, many sore arms have been produced; but nine out of ten were occasioned, or, at least, much aggravated, by the tightness of the clothes; by allowing the linen to stick to the sore; by scratching the pustule, and sometimes by emollient poultices. The experience we have had since January last in London, and in the country, does not agree exactly with Dr. Jenner's account concerning the state of the arms. He thinks some new applications of a caustic nature necessary in many cases to prevent secondary symptoms from the sores; but Dr. Woodville (Report, p. 155), my correspondents, and myself, have not found any want of applications on this account.

5. Concerning the important point of the certainty of the action of the cow-pox on the human constitution to produce unsusceptibility of taking subsequently the small-pox, I can only say at present, that I have inoculated many scores with small-pox matter after the vaccine disease, and never with the effect of exciting the small-pox. But I have had accounts sent to me, not of people taking the small-pox after the inoculated cow-pox, but of these taking the small-pox after the cow-pox in the casual way. I have, indeed, been desired to see even some of my own patients, who, I was acquainted, had taken the small-pox after the cow-pox; but these cases turned out to be either those in which the cow-pox had not in reality preceded, or they were cases of merely local affection from the inoculated small-pox.

With

With reſpect to the facts of other practitioners, I ſhall, at a future time, make ſome remarks on them to render their accounts conſiſtent with thoſe of Dr. Jenner, Dr. Woodville, and myſelf. In the mean time, I will not allow that any perſon's evidence is on this point much to be depended upon, unleſs he really knows what are characters of the cow-pox puſtule, and what are thoſe of the variolous, and ſome other common eruptions.'

By this remark, I do not mean to imply, that inferiority of natural judgment renders ſuch perſons' evidence of little weight, but I mean that any perſon whatever, who has not been accuſtomed to obſerve the appearances of certain eruptions, will ſcarcely be able to diſcriminate them. Therefore, as the vaccine poiſon by inoculation, eſpecially when the matter is dried, produces inflammation, a little tumour, and ſometimes puſtule, which are not the effects of the *ſpecific ſtimulation* of the matter, it is not ſurpriſing that reports ſhould be given of perſons taking the ſmall-pox after ſuch inoculation; and alſo of their taking the cow-pox more than once. Men of common accuracy in obſervation, by experience, will find out the miſtakes they have committed in this reſpect, but I am very much afraid that *all* have not the candour to acknowledge and rectify them, however honourable ſuch conduct is in the eſtimation of the beſt part of ſociety. It affords me great ſatisfaction to find my friend and correſpondent, Dr. Davis, of Bath, has diſplayed this honourable diſpoſition; I conjectured the caſe was as he has ſtated it, when he ſome months ago communicated it to me. (See *Medical and Phyſical Journal* for laſt month, p. 106). His conduct in this inſtance, although it is no more than I had a right to expect from my opportunities of knowing his ſtudious habits, I cannot help conſidering as a certain preſage of diſtinction in his profeſſion.

From the preceding general reſults, without entering into a more particular account, I think we may ſafely conclude, that the cow-pox inoculation is attended with advantages ſufficient to force its way ſpeedily into general practice; and that in courſe, it will ſuperſede, and ultimately extinguiſh, the ſmall-pox; but this concluſion is only made, provided no new facts ſhall ariſe, adverſe to the experience now poſſeſſed.

With regard to the ſecond object of this paper, Dr. Jenner, very uſefully to human ſociety, and very honourably to himſelf, firſt publiſhed ſome FACTS, which I thought it my duty, in common with other members of the profeſſion, to inveſtigate, and have laid before the public. Among theſe *facts*, the *fourth* and *fifth* were aſſerted in theſe terms:

IV. *A perſon having been affected with the ſpecific fever, and local diſeaſe, produced by the cow-pox poiſon, is liable to be again affected as before by the ſame poiſon; and yet ſuch perſon is not ſuſceptible of the ſmall-pox.*

V. *A perſon is ſuſceptible of the cow-pox, who has antecedently been affected with the ſmall-pox.*

Neither of theſe facts being ſupported by any analogy, a great part of the public ſeemed inclined to diſbelieve them; and not only inclined to diſbelieve theſe facts, but the credit of others was, for obvious reaſons, thereby weakened. It may be ſeen in my *Inquiry*, that I thought the aſſertion ſtood in need of confirmation, which I was not only unable to procure, but contrary evidence was obtained. Some of my correſpondents not only aſſerted that men were not affected more than once, but that the ſame cows had not been known to be affected more than once. It was alſo poſitively aſſerted by ſome, that a perſon is not liable to the infection of the cow-pox after going through the ſmall-pox (p. 49, *Inquiry*), "and I ſaw perſons pitted with the ſmall-pox, who had been much expoſed to the cow-pox without taking it." (*Ibid.* p. 50.)

Notwithſtanding my confidence in Dr. Jenner's evidence, I could not help pointing out, in the following words, what I apprehended was a ſource of error in both caſes: "The evidence for this fact (viz. IV.) to my apprehenſion only, proves ſatisfactorily, that the *local affection* of the cow-pox may occur in the ſame perſon more than once; but whether the *peculiar fever* alſo occurs more than once in the ſame perſon, from the cow-pox poiſon, does not appear certain, and muſt be determined by future obſervations, made with a peculiar view to this point." Farther; I was ſo diſſatisfied, that I wrote to Dr. Jenner to anſwer my query, Whether, in the inſtance of the cow-pox occurring more than once in the ſame perſon, it was certain that the ſpecific fever was preſent more than once? The Doctor very obligingly anſwered my letter, and ſays (ſee Dr. Jenner's letter, p. 99 of my *Inquiry*), "You may be aſſured, that a perſon may be repeatedly affected, *both locally and generally*, by the cow-pox; two inſtances of which I have adduced, and have many more in my recollection." But he very candidly adds, "Nevertheleſs, on this important point, I have ſome reaſon to ſuſpect that my diſcriminations have not been till lately ſufficiently nice."

With reſpect to fact V. I ſaid in my *Inquiry*, p. 49, "It ſeems ſufficiently authenticated, that people may have the cow-pox after they have had the ſmall pox; but it will require more nice attention to ſatisfy the query, Whether, in ſuch caſes, the cow-pox affects the whole conſtitution; or is only a local affection?" Subſequently to this obſervation, I find Dr. Jenner himſelf, from a theoretical conſideration, offers as a "conjecture, what experiment muſt finally determine, that they who have had the ſmall-pox, are are not afterwards ſuſceptible of the primary action of the cow-pox virus." (*Further Obſervations*, &c. by E. Jenner, M. D. &c. p. 32.)

I ſhall now relate the *trials* I have inſtituted, and the *obſervations* I have made, to obtain *determinations* with reſpect to theſe important queſtions of facts.

*Trials to determine whether or not Perſons be ſuſceptible of the* SPECIFIC COW-POX PUSTULE *and* FEVER, *who have undergone the Small-pox.*

The following four firſt-named gentlemen being engaged with me in phyſical inquiries, were deſirous to experience in their own perſons, the effects of the vaccine poiſon.

1. Mr. DANGERFIELD was inoculated in one arm by means of a puncture with a lancet ſtained with freſh, but dried matter, rendered fluid by ſteam, when inſerted. The other arm was inoculated by paſſing through the ſkin a bit of thread impregnated with matter.

On viewing the arms in three days time, that with the thread appeared inflamed, with a red, elevated, ſmall ſpot; the other arm which had been punctured, barely ſhewed a red ſpot. The punctures had ſmarted for about twenty-four hours, but no other effects were produced. Theſe red ſpots diſappeared in a few days.

In three weeks further the inoculation was again inſtituted, but with fluid lymph applied immediately from the puſtule of a patient preſent, to punctures in each arm. More ſmarting and more inflammation were produced by this inoculation than by the former. A ſmall quantity of pus was produced in the little red ſpots from the punctures in about ſix or ſeven days, but no diſorder aroſe in the whole conſtitution.

Mr. Dangerfield was next inoculated in one arm with *variolous* matter. In the evening of the day of inoculation, inflammation appeared, which increaſed to a greater degree and extent than from the vaccine inoculations. A ſmall phlegmonic tumour, in the part inoculated with variolous matter, continued for a fortnight, during which time it ſuppurated, and the ſore from it did not heal in leſs than three weeks further. There was no conſtitutional affection, but much pain was felt in the arm-pit in about five days from the inciſion.

2. Mr. POLLOCK was inoculated in each arm with a lancet armed with fluid lymph, immediately after taking it from a patient. A little ſmarting was felt for a day or two, and the parts inoculated were red for ſeveral days, but no puſtules aroſe, nor conſtitutional affection.

3. Mr. Perkins was inoculated by punćturing one arm with a lancet ſtained with recent *vaccine matter*, and the other arm was inoculated with variolous matter. A red ſpot was ſeen on each of the parts inoculated the day following, and an itching ſenſation, eſpecially from the vaccine matter, was experienced for a day or two. The parts remained elevated and inflamed a little for a few days further, and then got well without ſuppurating, or being attended by any general diſorder.

4. Mr. Armitage, whoſe conſtitution was fat and muſcular, was inoculated in each arm with a lancet ſtained with limpid vaccine matter, immediately on taking it from a patient preſent.

A ſmall red ſpot was obſerved the day following, and a little burning ſenſation was complained of. The red ſpots grew larger and larger for four or five days, and at length produced a ſmall, unequal, hard tumour, in which a little pus was afterwards generated; but the parts ſoon got well, without any attending diſorder of the whole conſtitution.

In a fortnight after this, each arm was inoculated with variolous matter: more inflammation aroſe, in a few days, than from the vaccine inoculation, followed by ſmall tumours, which ſuppurated. The parts inoculated remained ſore for more than a fortnight, but no feveriſh ſymptoms ever appeared.

5. G. P. a boy twelve years of age, who had gone through the cow-pox ten years before, was inoculated in one arm with recent dried vaccine matter, but rendered fluid by ſteam juſt before it was inſerted. The day following not ſo much as a red ſpot appeared on the part inoculated, nor had there been any uneaſy ſenſation. He was therefore inoculated a ſecond time, but with fluid lymph immediately from a patient.

The day after the ſecond inoculation, an itching ſenſation of the punćtured part was complained of, which continued for two or three days. The part punćtured had a ſmall, red, elevated ſpot upon it the day after the inoculation, which grew gradually larger for four or five days, and became a trifling phlegmonic tumour, but without any red ſurrounding areola. In a few days the little ſwelling ſubſided, but a red, and rather ſore ſpot, remained for a week longer. No diſorder of the whole conſtitution was perceived.

6. I was inoculated by Dr. Woodville, in one arm, with fluid vaccine lymph from a ſubjećt preſent. The punćtured part ſmarted a little all the remainder of the day of the inoculation, and alſo the day following.

In twenty-four hours a red ſpot on the inoculated part was ſeen, exaćtly like like that which is ſeen in the ſame time when either the vaccine, or variolous infection has taken effect, and increaſed for yet another day; but after this the redneſs vaniſhed, and no ſore was left.

I once accidentally punctured the back of my hand with a lancet which had fluid vaccine matter upon it. The conſequence was, a circumſcribed, very ſmall, red, hard tumour. This remained for a fortnight, then ſuppurated, and afterwards burſt. The part ſoon healed, but left a very ſmall ſuperficial cicatrix.

As belonging to this head, I mention that I have ſeen ſeveral inſtances of nurſes having ſmall, red, conical tumours on their lips and cheeks, and ſometimes hands, evidently from the application of cow-pox matter from the children under their care during the vaccine inoculation. Theſe little tumours ſometimes remained for ſeveral weeks, and a particle of pus was formed in them. They never were attended by any fever ſymptoms, nor by any ſurrounding erythematous areola. I here ſpeak of nurſes who had long before paſſed through the ſmall-pox.

I have no heſitation to refer the following caſes to this head of unſuſceptibility of taking the cow-pox, to having previouſly gone through the ſmall-pox.

A male ſervant to THOMAS KING, Eſq. about eighteen years of age, was brought up under circumſtances in which he could get no teſtimony to his having had, or *not* having had the ſmall-pox. Not having undergone this diſeaſe to his own knowledge, it was thought adviſable to inoculate him for the cow-pox, in order to reſiſt the ſmall-pox, with which his fellow-ſervant was ſeized. This I did on Wedneſday, the 23d of March, in one arm, with matter on a bit of thread.

4th day, Tueſday 26th. The parts inoculated had ſmarted for the firſt two days, and they were now red and a little elevated, as if the infection had taken.

6th day, Thurſday 28th. Inflammation had almoſt entirely gone off. Inoculated a ſecond time in both arms with matter from a different patient.

3d day of *ſecond inoculation,* Saturday 30th. The parts appeared inflamed.

6th day of *ſecond inoculation,* Tueſday, April 2d. Inflammation had diſappeared. Inoculated a *third time* with limpid fluid from a patient preſent, and with which matter I had excited the vaccine diſeaſe in ſeveral perſons.

7th day

7th day of *third inoculation.* The parts inoculated had inflamed and felt painful for two or three days, but were now well. Inoculated the patient a fourth time with *fmall-pox matter,* in both arms; from which a little inflamation arofe in both arms, but nothing more. This young man frequently vifited his fellow-fervant in the fmall-pox, at the Small-pox Hofpital, and often fhook hands with him, while under my care for the cow-pox inoculation.

In this cafe either the fmall-pox had already affected the conftitution, or fome other difpofition exifted, rendering it equally unfufceptible of the fmall-pox and cow-pox.

From Dr. Mitchell at Chatham, whofe report is now before me, I learn that there were feveral inftances of foldiers, to whom the cow-pox could not be communicated; and, although they had they had no recollection themfelves of having gone through the fmall-pox, it was moft likely they had really been affected by it.

If I had feen any inftance of genuine cow-pox puftule and fpecific fever in a conftitution which had previoufly fuffered the fmall-pox, I fhould have related it; but I ought to mention that fuch a cafe has fallen under the obfervation of Dr. Woodville (Reports, p. 52 and p. 143). I fhall never object to the teftimony of fo experienced a phyfician without more than ufual confideration; but I cannot avoid here obferving, that the evidence in his cafe of the patient having had the fmall-pox *when a child,* is merely that of the patient; and I fubmit to Dr. Woodville, whether or not that evidence is admiffible to build upon, now that we have the above unequivocal contravening cafes of the fact afferted. But I truft the Doctor will be lefs tenacious of this inftance, as he himfelf tells us that he failed to excite the vaccine difeafe, by inoculating feveral patients who were recovering from the natural fmall-pox. (Reports, p. 144.)

Whatever impreffion the above inftances may have made on my own mind, I do not expect they will produce conviction in the mind of every practitioner, *that it is a law of the animal economy to be rendered unfufceptible of the cow-pox fever and fpecific puftule, by undergoing the fmall-pox.*

Hence I find that my expectation of the hands of phyfic being ftrengthened by the poffeffion of a fure means of exciting an innocent fever is not realized (*Inquiry,* p. 81); but I feel fome confolation from the profpect of the new inoculation being more fpeedily introduced, by the removal of one obftacle, viz. the fears of many patients who have already paffed through the fmall-pox, that they would be liable to the cow-pox, if the diffufion of the the infection of it became extenſive by the vaccine inoculation. Another advantage ſuggeſted in my *Inquiry*, p. 92, is now, I think, greatly confirmed; namely, an advantage for thoſe who are not certain whether or not they have had the ſmall-pox, but poſſeſs ſo great a dread of this diſeaſe as not to be able to ſubmit to inoculation for it.

I congratulate ſuch perſons on the diſcovery of a teſt to which I apprehend the moſt timorous minds will ſubmit: for if the *ſpecific puſtule* and *fever* do not take place from the inoculation of the cow-pox poiſon, they may be aſſured, that *either they have already paſſed through the ſmall-pox*, or *their conſtitutions are not ſuſceptible of it.*

It now ſeems to me that the following facts are eſtabliſhed on the foundation of experience:

1. A conſtitution which has undergone the ſmall-pox is unſuſceptible of again undergoing the diſeaſe.

2. A conſtitution which has not undergone the ſmall-pox, but which has undergone the cow-pox, is unſuſceptible of undergoing the ſmall-pox.

3. A conſtitution which has not undergone the cow-pox, but which has undergone the ſmall-pox, is unſuſceptible of undergoing the cow-pox.

Now, if the variolous poiſon deſtroys the ſuſceptibility of the conſtitution to the future agency of this poiſon, in the reſpect of its producing the ſmall-pox—and if the cow-pox poiſon deſtroys the ſuſceptibility of the conſtitution to the future agency of the variolous poiſon, in the reſpect of its producing the ſmall-pox—and if the variolous poiſon deſtroys the ſuſceptibility of the conſtitution to the future agency of the vaccine poiſon, in the reſpect of its producing the cow-pox—it is demonſtrated, that the ſame ſtate of ſuſceptibility of the conſtitution with reſpect to the future agency of the variolous poiſon, is produced equally by the agency of the variolous poiſon, and by the vaccine poiſon; but, if the variolous poiſon produces unſuſceptibility of the conſtitution to the future agency of the vaccine poiſon, it is demonſtrable that the following fourth propoſition is true, viz.

4. A conſtitution which has undergone the vaccine diſeaſe is unſuſceptible of again undergoing that diſeaſe from the vaccine poiſon, becauſe a ſtate of unſuſceptibility with reſpect to the agency of the variolous poiſon is produced by the vaccine poiſon (2d *propoſition*), and a ſtate of unſuſceptibility with reſpect to the agency of the vaccine poiſon is produced by the variolous poiſon (3d *propoſition*);—but the ſtate of the conſtitution being the ſame in the two caſes, whether it be produced by the variolous, or vaccine poiſon, with reſpect to unſuſceptibility, it ſeems inevitably in courſe, that unſuſ

unfufceptibility of the conftitution to the future agency of the vaccine poifon is produced by the vaccine difeafe; and the demonftration, in courfe, could be given of the 1ft *propofition*, on the ground of the 2d and 3d *propofition*, that unfufceptibility of the conftitution to the agency of the variolous poifon is produced by the variolous difeafe, if this were not already proved by abundant experience. At a future time, however, I fhall relate the obfervations and experiments to confirm this *à priori* conclufion; firft, becaufe thefe proofs will increafe the validity of the 3d *propofition;* and fecondly, becaufe I do not mean to offer this demonftration as infallible, like mathematical.

From the preceding reafoning it may be imagined, that I confider the cow-pox and fmall-pox as the only varieties of the fame fpecies of difeafe, and that therefore, the name *variola vaccina* is appropriate, although I endeavoured to fhew that it was unjuft, and tended to miflead, by giving erroneous notions (*Inquiry*, p. 108). But it muft here enter into our contemplation, that the fame ftate of an animal, or other fubftance, in a certain refpect, may be produced by very different things, and the phenomena attending their agency, may be very different from one another. It is fo in the inftances under confideration; and farther, in order to eftablifh refembling things to be *varieties of the fame fpecies, we ought to be able to trace them to one common origin,* or to fhew that they all agree in what fhould be reckoned effential properties. Now, hitherto it has not appeared that the cow-pox has arifen from the fmall-pox, or the fmall-pox from the cow-pox. If it be faid, that in fome of the eruptive cafes of the cow-pox the puftules could not be diftinguifhed from the fmall-pox, it fhould be confidered, that it has not been yet fhewn that in any cafe the fmall-pox has changed into the cow-pox; that the cow is fufceptible by inoculation of the cow-pox, by inoculation of the matter of the cow-pox from the human fubject; and that the puftules refembling the fmall-pox, which occur in the cow-pox, afford matter which, I believe, produces in fome cafes (although perhaps not in fo great a proportion as originally) the cow-pox in its ufual mild way, viz. a puftule in the inoculated part only, and a flight fever. Hence I humbly am of opinion (but fubmit the queftion to the decifion of fcholars), that the denomination *variola vaccina* is a tranfgreffion of the law in philology, and repugnant to found logic.

Extended as this paper is, much beyond the limits propofed, I cannot confine to myfelf the gratification from the Reports of the *New Inoculation*. I fhall only mention, however, one or two of them.

The fenfation excited on the continent by the vaccine inoculation, has been been much more confiderable than even in our own ifland, as I learned, firft from Dr. Marcet, and fince, by a letter from Dr. Peschier. At Vienna, Dr. Ferro inoculated two of his own children with vaccine matter, which I tranfmitted; and next, Dr. De Carro inoculated two of his own children. An accurate journal of thefe two laft cafes was kept by Dr. de Carro, which he has had the complaifance to communicate to me through the hands of Dr. Pefchier. The above patients had the vaccine difeafe in the ufual mild way that they have had it in England, and were inoculated fubfequently for the fmall-pox, but without taking that difeafe.

It is expected that Dr. Frank will adopt the new inoculation, as it is likely to be generally done at Vienna.

I expect reports from Portugal, and other parts of the Continent.

When matter is to be kept for a long time, I preferve it on thread, which I enclofe in a bottle filled with hydrogen gas, or nitrogen gas, quite free from moifture.

In Scotland the new inoculation has not been lefs fuccefsful. Dr. Anderson, of Leith, informs me he has inoculated above eighty perfons; that Dr. Duncan, and others, have begun the practice at Edinburgh; and that it has been introduced in Dundee, Paifley, and Dalkeith.

If the vaccine inoculation proceeds with equal mildnefs as it has done the laft four months, doubtlefs the variolous incifion muft in no remote period be fuperfeded. And if fuch an event fhould take place, pofterity will behold with amazement, the prejudices and inattention of their predeceffors to the application of a fact in practice, by which a formidable and loathfome difeafe was extinguifhed—a fact well known, time immemorial, to almoft every farmer in half a dozen counties of England, but neglected till Jenner had the courage to indicate the advantages of it to fociety. If I were to name a parallel inftance of inattention or prejudice, it fhould be the neglect of inoculation for the fmall-pox till it was introduced into England from Conftantinople, although it had been practifed time immemorial in the Barrozzo mountains, on the frontiers of Gallicia, in the fame rude manner that it is at this day.*

To

* This intelligence was communicated to me by a Portuguese nobleman, whose opportunities of information and accuracy authorize me to mention the fact: but an attested account from some of the inhabitants of those mountains is intended for me.—See a book written by *Jacobus a Castro Sarmento,* in which, I am told, this fact is asserted.

